# Cannabinoids, their cellular receptors, and effects on the invasive phenotype of carcinoma and metastasis

**DOI:** 10.1002/cnr2.1475

**Published:** 2021-07-26

**Authors:** Judah Glogauer, Jonathan Blay

**Affiliations:** ^1^ Michael G. DeGroote School of Medicine McMaster University Waterloo Regional Campus Kitchener Ontario Canada; ^2^ School of Pharmacy University of Waterloo Waterloo Ontario Canada; ^3^ Department of Pathology Dalhousie University Halifax Nova Scotia Canada

**Keywords:** biased signaling, cannabinoids, CB1, CB2, GPR55, invasion

## Abstract

**Background:**

The morbidity and mortality of cancer are significantly impacted by the invasive and metastatic potential of particular subgroups of malignant cells within a tumor. The particular pre‐metastatic properties of cancerous cells are thus a critical target for novel therapeutics in the oncology field. Cannabinoid molecules have been investigated in recent years in the context of invasion and metastasis of various malignancies, with varying effects reported in the literature.

**Recent Findings:**

There was substantial variability in the findings reported by the literature of the effects of cannabinoid molecules on cancer cell invasion and metastasis. These effects varied depending on which ligand and which of the CB1, CB2, or GPR55 receptors were investigated. These findings suggest a role for the phenomenon of biased signaling in explaining the diversity of effects of cannabinoid molecules on cancer cell invasion.

**Conclusion:**

While substantially more investigation is required into the effects of cannabinoid molecules on cancer cell invasion and metastasis, we describe in this review the significant diversity in the responses of cancer cells to cannabinoid molecules in terms of their invasive and metastatic capacities.

## 1 INTRODUCTION

The ability of cancer cells to invade neighboring tissues and subsequently metastasize to distant sites is the largest contributing cause of the morbidity and mortality of malignancy.^1^ It has been estimated that more than 67% of deaths from cancer are due to the spread of the malignant cells rather than the primary tumor itself.^2^ Identification of novel therapeutic approaches to slow or halt the spread of malignant cells is therefore of immense interest to both clinicians and scientists. Much of this research has examined the effects of endogenous and exogenous (ingested) molecules on the migratory and invasive activity of individual cancer cells. Due to the highly prevalent usage of cannabinoids in palliative care^3^ and recent legalization of cannabis recreational use across multiple countries, this source of bio‐actives has become a focus for those seeking novel therapeutics to prevent tumor cell migration, invasion, and metastasis.^4^

Significant research has already been conducted on cannabinoids in the context of cancer cell proliferation,^5^ tumor formation,^6^ and angiogenesis.^7^ However, the properties of cannabinoid molecules in the context of cancer and non‐malignant cell migration, invasion, and metastasis remain poorly understood. Numerous studies report conflicting results, including be *enhanced* migration mediated through phosphatidylinositol 3‐kinase (PI3K),^8^ *increased* migration through mitogen‐activated protein kinase (MAPK) pathways,^9^ *decreased* migration through modulation of matrix metalloproteinase (MMP) activity^10^ and *decreased* migration through a cannabinoid‐receptor‐independent mechanism.^11^

Studies investigating these diverse phenomena indicate that cannabinoid signaling and its subsequent effects on cell migration and invasion are not as straightforward as would be anticipated from a single type of molecule activating one or two downstream signaling transduction cascades. Cannabinoid molecules exhibit significant diversity in their structures, functions, and downstream effects in the context of cell migration and invasion, due to a phenomenon known as biased signaling.^12^ This phenomenon, which will be explored here through the lens of cannabinoid effects on cell migration and invasion, leads to significant differences in the observed effects of cannabinoids.

We will first consider the major players in the endogenous system that is exploited by cannabinoids that are ingested or applied therapeutically, beginning with the receptors involved and outlining the cellular signaling pathways that ultimately determine the multiple outcomes in research experiments involving cannabinoids and cancer cell invasion.

## 2 THE ENDOCANNABINOID SYSTEM

The endocannabinoid system, the endogenous network on which exogenous cannabinoids act, is composed of a set of G‐protein‐coupled receptors (GPCRs) and their associated cannabinoid ligands. These control a variety of physiological states and cellular functions, including cell proliferation, migration, and survival.^13^, ^14^

### 2.1 Cannabinoid receptors

The major receptors are the CB1 and CB2 receptors, with both receptors being well studied in a number of pathological states, including malignancy.^14^ The most abundant of these receptors in the CNS is the CB1 receptor, primarily present on axon terminals and pre‐terminal axon segments. CB2 is expressed significantly less in the CNS, and is more important in the immune system and gastrointestinal tract, but has been observed primarily on microglia and various blood vessels.^15^ Both of these cannabinoid‐binding GPCRs, acting through different second messengers, possess the ability to initiate signals through multiple pathways responsible for diverse cellular functions.^16^, ^17^

More recently, several other receptors have received attention in the literature as potential members of the endocannabinoid system, including GPR55 and GPR18. GPR55, an atypical cannabinoid receptor, is a typical seven‐transmembrane GPCR and has been identified as a receptor for the endogenous cannabinoid ligand L‐A‐lysophosphatidylinositol as well as exogenous cannabinoids such as cannabidiol.^18^ GPR18 is another GPCR that normally binds N‐arachidonoyl glycine and has shown to be activated by Δ‐9‐THC.^19^ The endocannabinoid system consists of a large number of receptors and associated ligands, including the aforementioned receptors, as well as others, including GPR119,^20^ various members of the PPAR family,^21^ and others. As this review focuses on the receptors of the endocannabinoid system that are implicated in cancer cell invasion, the focus will be placed on CB1, CB2, as well as GPR55 and GPR18, receptors that have been the focus of research examining the effects of cannabinoids on cancer cell invasion.

### 2.2 Ligands of the endocannabinoid system

The ligands of the endocannabinoid system are numerous and varied, consequently this paper will only focus on those ligands that have been investigated in the context of cancer cell invasion. Cannabinoid ligands can be subdivided into endogenous and exogenous forms, with exogenous cannabinoids including the well‐publicized Delta‐9‐ tetrahydrocannabinol (or THC), and endogenous cannabinoids being endogenous lipids that activate signaling pathways downstream of cannabinoid receptors.^21^ The two most commonly studied endocannabinoids are arachidonoyl ethanolamide (also known as anandamide) and 2‐arachidonoylglycerol, both having precursors present within cell membranes.^21^ These two endocannabinoids, despite their similarities, have different affinities for CB1 and CB2, indicating inherent differences for endocannabinoids in terms of their efficacy in activating different receptors.^22^, ^23^ These ligands have been shown to act on major cannabinoid receptors, as well as other receptors that have been considered part of the endocannabinoid system including receptors of the PPAR family.^24^ Other endocannabinoid molecules include O‐arachidonoylethanolamine (also known as virodhamine), N‐arachidonoyldopamine (NADA), 2‐arachidonoylglycerol ether (2‐AGE).^25^ While these endogenous lipids may also be considered part of the endocannabinoid system, Anandamide and 2‐arachidonoylglycerol have been identified as being the primary members,^21^ and have been implicated in the context of cancer biology.^26^, ^27^ This paper will further review the effects of key endogenous and exogenous cannabinoids in the context of cancer cell invasion.

### 2.3 Degradation enzymes of the endocannabinoid system

A key regulatory element of the endocannabinoid system is the group of enzymes responsible for the degradation of endocannabinoids ligands. Anandamide is degraded through hydrolysis within the brain and the spinal cord by fatty acid amino hydrolase,^21^ an enzyme also responsible for the degradation of other amino acids.^28^ This enzyme may prove to have relevance in the context of cancer invasion, as the inhibition of this enzyme has shown to diminish the invasiveness of colon cancer cells.^29^ Anandamide and 2‐arachidonoylglycerol are both hydrolyzed by COX‐2.^30^ 2‐arachidonoylglycerol is also broken down by a number of different hydrolytic enzymes including monoacylglycerol lipase.^31^ See Fowler et al^31^ for an excellent overview of the hydrolytic enzymes of the endocannabinoid system.

### 2.4 The phenomenon of biased signaling

Studies examining these receptors and ligands have identified diverse responses exhibited from the same receptor with the binding of different ligands. This multitude of responses exemplifies the phenomenon of biased signaling, whereby the same receptor can exert multiple effects, depending on the individual ligand. Biased signaling, also known as functional selectivity, occurs due to the various conformational states that GPCRs can adopt.^32^, ^33^ The binding of a ligand to the receptor causes the stabilization of the receptor into a particular conformational shape, thus determining a particular cascade of events leading to downstream intracellular signaling. Depending on which shape the receptor takes, different second messengers will be activated and different downstream processes can be initiated. Thus numerous signaling pathways can be activated by the same receptor. This phenomenon has far reaching implications for pharmacological interventions in the context of disease, as different treatments could potentially target the same receptor, while still achieving different goals.

The significance of this phenomenon can be illustrated by a study by Atwood et al conducted in 2012 to examine CB2 receptors in a mouse model.^4^ These researchers found that the receptor could inhibit voltage‐gated sodium channels when activated with one of its ligands, CP55,940, but not when another of its ligands was bound, WIN55,212–12. This was not merely a case of only one ligand being capable of binding, as both ligands induced hyperpolarization of the same cell line in a separate experiment.

### 2.5 Other sources of complexity in signaling

There are differences in the degree of biased signaling between different members of the endocannabinoid system. For example, CB1 has been shown to display significant promiscuity relating to which G‐Proteins it can activate. While both CB1 and CB2 have noted affinity for Gα_i_ type G Proteins, CB1 displays activity of both Gα_s_‐ and Gα_q_‐dependent signaling depending on the cell line used or experimental conditions, whereas CB2 does not appear to display this level of promiscuity.^34^ While this could be a function of which G proteins are being investigated, with lesser promiscuity being displayed by CB2 as a result of unstudied proteins, current evidence supports the differences noted. What is most evident from the current literature is that numerous downstream pathways can be initiated by the same receptor, depending on the conditions studied, as well as the ligand that is utilized.

In some cases, the signaling pathway that is responsible for an observed phenotypic change can be transduced by different mediators. For example, in multiple publications, researchers have found CB1 activation of ERK1/2 to be downstream of different effectors,^8^, ^35^, ^36^ with all three publications noting similar observed outcomes with very different pathways being initiated. CB1 has been found to also activate members of the receptor tyrosine kinase (RTK) family to cause downstream effects, particularly in the activation of ERK1/2 through VEGFR^37^ and EGFR.^38^

The complex effect of cannabinoids in the context of cellular signaling has substantial implications for both studying and understanding cannabinoid regulation of cell migration and malignant cell invasion, based upon disease exploitation of the endocannabinoid system.

## 3 CANNABINOIDS, CANCER CELL INVASION, AND METASTASIS

There are two major phenomena in tumor progression of solid carcinomas: (i) invasive growth involving the acquired ability of cancerous cells to both migrate and penetrate through tissue barriers and walls, and (ii) metastatic spread utilizing the body's lymphatic drainage system or circulatory system.^39^ A neoplasm that does not have these abilities is referred to as carcinoma in situ^40^ and is typically not in itself threatening without further evolution of the disease. Thus, it is the invasion and metastasis, including cellular migration, that are the fundamental causes of morbidity and mortality, and which will be our focus in this review.

### 3.1 Cannabinoids, the epithelial‐mesenchymal transition, and invasion

The process by which cells acquire the ability to invade neighboring tissues was first referred to by Elizabeth Hay as the “epithelial‐mesenchymal transformation” in the context of embryogenesis.^41^ In the context of neoplasia, the phenomenon is commonly known as the “epithelial‐mesenchymal transition” or “EMT,” to reflect both the reversibility of the process and the distinction between the process itself and the process by which cells become neoplastic. As detailed by Kalluri and Weinberg, EMT occurs in three distinct biological settings, with only the neoplastic condition being necessarily pathologic.^42^ This is the context on which we will focus.

Researchers such as the aforementioned Kalluri and Weinberg^42^ have sought to explain the process by which cancer cells acquire the ability to invade neighboring structures, with EMT being proposed as the primary mechanism. A study of the invasive front of tumors observed that many of the leading cells have mesenchymal phenotypic markers such as α‐SMA, FSP1, and vimentin.^43^ It is currently unclear which are the primary factors that initiate the change from an epithelial phenotype to a mesenchymal one. It may be that genetic or epigenetic changes that occur during the early stages of neoplastic transformation render the cells sensitive to EMT‐related signals that exist within the stroma of the local microenvironment.^43^ Certain signals derived from the tumor stroma have been shown to activate EMT‐related transcription factors, such as TGF‐β acting through up‐regulation of the transcription factor Snail.^44^

Once EMT‐related transcription factors are activated, pleiotropic changes occur, activating the full EMT program and causing the neoplastic cell to (i) down‐regulate adherence proteins that would otherwise keep it anchored to the adjacent basement membrane, (ii) release proteolytic enzymes that allow degradation of the basement membrane and extrusion through to the tissue spaces, (iii) degrade subsequent extracellular matrix and escape into a neighboring tissue, organ, lymphatic duct, or blood vessel.^45^ While the full network of signals and transcription factors involved in this transition remains to be elucidated, significant progress has been made towards identifying individual proteins that play key roles in EMT, with many of these proteins being considered as future targets for therapeutics.

Recent evidence has shown an influence of cannabinoids on EMT‐related molecular markers (See Table 1 for a summary of the effects of Cannabinoids on EMT related markers as well as cancer cell invasion). Martínez‐Martínez et al treated HT‐29 cells, which over‐express cannabinoid receptor CB2, with the CB2 agonist JWH 133 and measured membrane levels of E‐Cadherin and intracellular levels of SNAIL1, a transcription factor linked with EMT.^46^ They found significant loss of membrane E‐cadherin and higher levels of SNAIL1. The researchers also found a link between CB2 expression, SNAIL1 levels, and increased incidence of lymph‐node‐positive disease in human patients, suggesting a mechanistic and potentially prognostic role for levels of CB2 and EMT.

**TABLE 1:** Summary of the effects of cannabinoid ligands on cancer cell invasion and metastasis

Receptor	Cancer type	Ligand	Effect	References
CB_2_	Colon cancer (HT 29 cells)	JWH 133	Increased metastasis	Martínez‐Martínez et al^46^
CB_1_	Endometrial cancer	Δ‐9‐tetrahydrocannabinol	Decreased invasion	Zhang et al^47^
CB_1_ and CB_2_	Chronic myelogenous leukemia	CB_1_ and CB_2_ Agonists, CB_1_ Antagonist	Agonists = Decreased potential for invasion Antagonist = Increased MMP9 and MMP2	Gholizadeh et al^48^
CB_1_ and CB_2_	Gastric cancer	WIN55,212‐2 AM251 and AM630	WIN55,212‐2‐Decreased invasion, AM251 and AM630‐Reversal of decreased invasive effect	Xian et al^49^
CB_1_	Prostate cancer	WIN55,212‐2 2‐arachidonoylglycerol	WIN55,212‐2‐Decreased migration, 2‐arachidonoylglycerol‐Decreased migration	Nithipatikom et al^50^
CB_2_	Hepatocellular carcinoma	ACEA CB65	ACEA‐Decreased invasion CB65‐Decreased invasion	Pourkhalili et al^51^
CB_2_	Breast cancer	JWH‐015	Decreased invasion	Elbaz et al^52^
CB_2_	Non‐small cell lung cancer	None (Knockdown of CB_2_)	Decreased invasion with knockdown of CB_2_	Xu et al^53^
GPR55	Pancreatic cancer and Liver cancer	Lysophosphatidylinositol O‐1602 AM251	Increased migration	Paul et al^54^
GPR55	Breast cancer (MDA‐MB‐231 cells and MCF‐7 cells)	L‐alpha‐lysophosphatidylinositol	Increased migration	Ford et al^55^
GPR55	Breast cancer	Lysophosphatidylinositol	Increased migration	Andradas et al^56^
GPR55	Colon cancer	Lysophosphatidylinositol	Increased migration	Kargl et al^57^
GPR55	Breast cancer	Lysophosphatidylinositol	Increased migration	Zhou et al^58^

Other publications have found interactions between cannabinoids and EMT‐related proteins and transcription factors, but with sometimes contrasting effects depending on the cannabinoid receptor involved. Zhang et al showed that incubating endometrial cancer cells with Δ‐9‐tetrahydrocannabinol, investigated in this context as a CB1 agonist, decreased the levels of MMP9 in an endometrial cancer model.^47^ MMP9 is a MMP that is key to the progression of endometrial cancer, degrading extracellular matrix and leading to the possible extravasation of malignant cells into the bloodstream or lymphatics to enable metastatic spread. The downregulation of MMP9 is known to decrease cancer cell migration and invasive potential. The researchers also found that Δ‐9‐THC decreased EMT activity in the endometrial cancer cells, significantly impairing mobility and invasion.^47^

The difference between the implications of these two studies^46^, ^47^ in terms of the possibly opposing effects of different cannabinoids, is a common theme in the present literature. Gholizadeh et al found that administering CB1/CB2 agonists to K562 chronic myelogenous leukemia cells decreased MMP9 and MMP2 whereas administration of a CB1 antagonist led to the opposite effect.^48^ While the focus here was not on the overall process of EMT, a significant effect of cannabinoid administration on metalloproteinase activity was observed.

Other publications have shown an indirect mechanism by which tumor cells can experience an increase in metalloproteinases, mediated through cannabinoids. Sailler et al found that as tumors progress to a metastatic phenotype, concentrations of the endocannabinoid 2‐arachidonylglycerol increased.^59^ They also showed that 2‐arachidonoylglycerol mediated the phenotypic change of macrophages towards a typical tumor‐associated macrophage (TAM) phenotype, which is associated with increases in MMP secretion.^59^ The authors hypothesized that this mechanism may contribute to the tumor's ability to metastasize.

### 3.2 Cannabinoids and metastasis

Invasion and metastasis are by nature overlapping and sequential, existing on a phased continuum. For malignant cells to be able to enter the local lymphatics or blood capillaries, invasion through the extracellular and structural barriers of the surrounding tissue is necessary. After the transformational phenotypic process of EMT, metastatic tumor cells then travel through the systemic circulation or lymphatic system to arrive at a potentially hospitable area to attempt to colonize the new tissue and begin the formation of a secondary tumor.

The successful metastasis of a tumor to a distant site is a highly complex process, but known to depend on the presence of local environmental factors that favor residence, a concept first noted in the “seed and soil” hypothesis developed by Stephen Paget in the 1800s.^60^ Cannabinoids have been shown to have varying effects on the ability of tumors to establish a metastasis, although from much of the data it is unclear at which stage of metastasis this effect is occurring. Qamri et al noted that mice with metastatic breast cancer showed significant reduction of lung metastases when treated with synthetic cannabinoid receptor agonists.^61^ The anti‐metastatic effects of this treatment were abrogated when cannabinoid receptor antagonists were also administered, indicating that the observed phenomenon was cannabinoid receptor‐mediated.^61^

Cannabinoids also influence another key process in the development of the secondary tumor, local angiogenesis that is required to maintain the growth of the tumor. Occurring after a key step known as “the angiogenic switch” and enabled principally by vascular endothelial growth factor (VEGF), it is required to ensure that the tumor is able to acquire vital nutrients and oxygen essential for its survival.^62^ Cannabinoids have been shown to inhibit angiogenesis in a variety of different oncological models,^63^, ^64^ indicating a potential role for cannabinoids as anti‐metastatic agents.

Cannabinoids act on the metastatic process at various stages. This, together with the complexity of cannabinoid signaling at the cellular level, means that there are significant challenges in establishing whether cannabinoid receptor agonists or antagonists might be of value for the treatment or support of cancer patients in late‐stage, metastatic disease. However, the convergence of evidence indicates that cannabinoid interactions with cancer in the process of invasion and metastasis are a promising area for detailed study, with particular attention to receptor subtype.

## 4 CANNABINOID RECEPTOR ACTIVATION AND CANCER CELL INVASION

### 4.1 CB1 receptor activation and cancer cell invasion

When studying the effects of cannabinoids in the context of cellular changes in neoplastic progression, determining which receptor is implicated in a particular pathway can be a vital first step. For the CB1 receptor, G_i_ inhibitory proteins might participate in influencing cell migration, and based upon past experimentation investigating CB1 signaling through Gi protein second messengers,^65^ it was proposed that CB1 receptor signaling may have a major influence on cancer cell migration. In a 2000 study by Song and Zhong, a modified Boyden chamber assay was performed utilizing HEK cells transfected with the human CB1 gene and treated with CB1 agonists HU210, WIN55,212‐2, and anandamide.^65^ The results showed migratory responses occurring at least in part through CB1‐mediated intracellular signaling pathways, and this effect could be abrogated using a selective CB1 antagonist SR141716A. This discovery of the relevance of CB1‐mediated signaling led to study of the mechanism by Yao et al, who examined the presence of both CB1 and CB2 receptors in the human placenta and identified that the treatment of human amniotic placental cells with Δ9‐THC decreased cell migration through the regulation of MMP9 and MMP2 metalloproteinases.^66^ CB1 signaling likely plays a role in cell migration in both physiologic and pathological contexts including malignancy. Numerous papers have examined the effects of CB1 activation on cancer cell migration and invasion and found conflicting results. Xian et al treated gastric cancer cells with WIN55,212–2, an established CB1 agonist.^49^ Cell invasion was significantly decreased, and this effect was attenuated utilizing both AM251 and AM630, selective CB1 and CB2 receptor antagonists respectively. This effect of WIN55,212–2 through cannabinoid receptors CB1 and CB2 was found to be at least partially regulated through the reduced expression of MMPs and VEGF. An additional study performed by Nithipatikom et al identified the CB1 receptor as a negative regulator of prostate carcinoma cell migration through the inhibition of RhoA, a key GTPase involved in the cell migratory response.^50^ Treatment of prostate carcinoma cells with the selective CB1 agonist WIN55,212–2 led to a decrease in migration with a significant diminishing of RhoA activity, while treatment with CB1 selective antagonist AM251 showed a significant increase in RhoA activity. An artificial increase in 2‐arachidonoylglycerol, an endogenous CB1 and CB2 agonist, significantly diminished the migratory capacity of the same prostate cancer cell line through the inhibition of adenylyl cyclase. Conversely, decreasing the intracellular level of 2‐arachidonoylglycerol significantly increased migration of the same cells. This supports the view that CB1 plays a significant role in mediating cancer cell migration through the downregulation of cell migration. However, further studies have found an opposite effect. Li et al examined the role that the microRNA miR‐1273 g‐3p played in the migration of LoVo cells, through a CB1‐mediated mechanism.^67^ When the levels of this miRNA were decreased, cell migration was significantly decreased, and when *CNR1*, the CB1 gene, was knocked down, loss of migration was then attenuated. Other studies has shown increased migration through a CB1‐mediated mechanism in physiologic conditions, including the chemotactic migration of corneal cells and the differentiation of cells within the hematopoietic system.^68^, ^69^ While this contradictory evidence does not negate the previously observed effects, it does illustrate the failure of a one‐receptor‐one‐mechanism explanation for cannabinoid receptor signaling in the context of cancer cell migration, and emphasizes the importance of the concept of biased signaling and that the observed effect of CB1 ligands on cancer cell migration is context dependent. Future research efforts on cannabinoid signaling in the context of cancer cell migration require consideration of biased signaling and the resulting diversity of possible effects.

### 4.2 CB2 receptor activation and cancer cell invasion

Although the CB2 receptor also shows complexity in its association with migration signaling pathways, it appears to display less phenotypic diversity than the CB1 receptor in the context of cancer cell migration. In a 2012 study by Pourkhalili et al, human hepatocellular carcinoma cells were treated with either CB1 and CB2 receptor agonists and the invasion capacity was measured through the use of a cellular invasion assay.^51^ While the CB1 agonist ACEA was found to inhibit cell migration only within certain concentration ranges, the CB2 agonist CB65 was found to markedly decrease cell migration at all tested concentrations.^51^ The changes in migration were accompanied by notable decreases in MMPs under the influence of both the CB2 and CB1 agonists, but the concentration related differences remained; which may explain the decreased migration observed when using this invasion assay as the cells would be unable to penetrate the Matrigel® barrier. As described earlier, other workers have found similar decreases in the concentration of MMPs, particularly MMP9, such as the aforementioned study performed by Zhang et al who found a significant decrease in MMP‐9 when incubating endometrial cancer cells with Δ9THC.^47^

Similar CB2‐mediated decreases in cancer cell invasion have been observed elsewhere. Elbaz et al found that activation of CB2 with a CB2‐specific agonist (JWH‐015) inhibited EGF and IGF‐1 mediated invasion and migration in both estrogen receptor‐positive and‐negative breast cancer cells.^52^ CB2 receptor agonist (JWH‐015) stimulation prevented the activation of EGFR, IGF‐1 receptor and their downstream targets, including various MMPs, STAT3, AKT, ERK, NF‐kB and others. IGF‐1R over‐expression in breast cancer cell lines has been shown to be associated with anti‐apoptotic mechanisms, and increased cancer cell invasion, specifically with up regulation of MMP's and activation of EMT in Estrogen receptor alpha positive cells. As a result, IGF‐IR‐over‐expressing cancer subtypes tend to have poorer prognosis. The effects of CB2 activation on down‐regulating IGF‐I, as well as EGFR, signaling show a significant decrease in invasive potential.

Further research points to the converse possibility of pro‐invasive effects of CB2 signaling, similar to the observations of biased signaling in CB1 signaling. Xu et al demonstrated a positive relationship between CB2 expression and poor prognosis in NSCLC cells in clinical samples.^53^ In addition, siRNA knockdown of CB2 of various cell lines in vitro was found to drastically decrease the invasive and proliferative potential of malignant cells, specifically through downregulation of PI3K/Akt/mTOR, decreasing phosphorylation of Akt and mTOR. In addition, knockdown of CB2 produced a pro‐apoptotic effect in vitro through modulation of the Bcl2/Bax axis, indicating a pro‐survival effect of CB2 signaling. Other publications have found other pro‐tumorigenic and pro‐invasive effects of CB2 signaling in other cancers, including colon cancer^70^ and breast cancer.^71^

The biased signaling phenomenon therefore appears to be significant in the context of CB2 signaling in the context of cancer cell invasion. Depending on the context, CB2 signaling can induce or limit cancer cell migration. We do however have degrees of knowledge about biased signaling between CB2 and CB1 signaling. For CB1 investigations, there is significant data involving various ligands, whereas most available data in the CB2 realm focuses on siRNA knockdown. While some of the pro‐invasive data has utilized CB2‐selective agonists such as JWH‐133, more research will be required to determine the effects of biased signaling on CB2 mediated cancer cell invasion.

### 4.3 GPR55 receptor activation and cancer cell invasion

While cannabinoid signaling studies have mostly focused on transduction through CB1 and CB2 receptors, several other GPCRs have been hypothesized to be part of the endocannabinoid system and therefore available for intervention, with a number of endogenous and exogenous cannabinoids having binding and signaling potential. Among these receptors is GPR55, found to be a receptor for a number of cannabinoid ligands including lysophosphatidylinositol (LPI) and the synthetic cannabinoid AM251.^72^, ^73^ GPR55 has been shown to activate the Rho family of GTPases and has been indicated to have roles in cell migration outside of cancer, including in osteoclasts.^74^

However, as consideration of this receptor as a member of the endocannabinoid system has a relatively recent history, the number of publications focusing on the effects of cannabinoids on GPR55‐mediated cancer cell invasion is fewer than for CB1 and CB2. Nevertheless, several reports have noted changes in cancer cell invasion and migration in response to the binding of cannabinoid ligands to GPR55 receptors. In examining cell migration, Paul et al found that treatment of Panc‐1 pancreatic cancer and HepG2 liver cancer cells with atypical cannabinoid compound O‐1602 (a further GPR55 agonist) and LPI, led to the emergence of long filopodia and lamellipodia, both markers of cellular migration.^54^ In addition, treatment with AM251 treatment, also an agonist of GPR55, was shown to induce cell motility through ERK1/2 phosphorylation and intracellular Ca^2+^ release. Such findings point strongly to a promigratory effect of GPR55 receptor activation. A separate study performed by Ford et al found that when highly metastatic MDA‐MB‐231 breast cancer cells were exposed to LPI, cell chemotaxis towards serum was significantly enhanced.^55^ In the same study MCF‐7 cells (which have low metastatic potential and ordinarily do not express high levels of GPR55) were made to over‐express GPR55 before being exposed to LPI and were then found to have a significant robust migratory and invasive response. Knockdown of GPR55 with siRNA was found to abrogate LPI‐induced migration when compared to controls with an empty vector. Andradas et al also found an increase in metastasis in MDA‐MB‐231 cells when treated with LPI, mediated through activation of RhoA and a transcription factor known to be related to metastatic potential, ETV4/ PeA3.^56^ In addition to these findings, the researchers found that levels of GPR55 in triple‐negative breast tumors correlated with poorer prognosis and likelihood of metastasis.

Other papers have shown an increase in cancer cell migration mediated through GPR55. Kargl et al found LPI‐induced decreased adhesion and increased migration through GPR55 in HCT116 metastatic colon cancer cells as well as highly increased levels of LPI present in the serum of colon cancer patients.^57^ Zhou et al found an increase in migration of MCF‐7 and MDA‐MB‐231 cells with LPI, and further implicated the MLCK/p‐MLC pathway and HBXIP/p‐ERK1/2/Capn4, transcription factors that had been noted to be involved in breast cancer migration and metastasis.^58^ They found that LPI induced migration through GPR55, inducing the upregulation of HBXIP, p‐ERK1/2, MLCK, p‐MLC and Capn4, and that knockdown of GPR55 led to a decrease in the levels of all of these factors, indicating that GPR55 could be inducing its increased invasive effects through these transcription factors. Furthermore, the researchers found that GPR55 expression was correlated with the number of lung metastases in mice with breast cancer, with siRNA knockdown decreasing the number of metastases in mice injected with MCF‐7 cells.

Interestingly, compared with studies examining CB1‐ and CB2‐induced migration of cancer cells, the variability of responses, with activation inducing both pro‐ and anti‐migratory effects has not been convincingly demonstrated in GPR55 studies. The convergence of evidence appears to support a purely pro‐migratory phenotypic effect. However, more research will need to be conducted before this can be concluded, as the number of publications focusing on this phenomenon is still comparatively small.

## 5 OVERALL PERSPECTIVE

The effects of cannabinoids on cancer cell invasion are varied depending on (i) cancer cell type, (ii) receptor that is activated, (iii) the particular ligand used, and (iv) the context of the exposure; with phenotypic diversity being displayed even with these variables being held constant. The responses to CB1 and CB2 receptors, and to GPR55, provide an array of possible outcomes. Furthermore, the phenomenon of biased signaling, often evident when observing the effects of ligands on GPCRs, likely plays a role in determining the final response. Notably, another possible explanation that may be responsible for the conflicting responses that have been observed is the “entourage effect.” This effect was initially detailed by the lab of Raphael Mechoulam to explain the varied results of experiments involving cannabinoids in various systems across the human body.^75^, ^76^ The entourage effect details the synergistic effect that unrelated metabolites and other compounds that do not bind to the cannabinoid receptors can have in combination with cannabinoid molecules.^77^ This effect has been implicated in the differential responses that cancer cells can have when exposed to cannabinoid molecules in a number of different cancer models.^78^, ^79^ This effect has the potential to account for the varied responses that occur when cancer cells are stimulated by cannabinoid molecules. However, significantly more research must be conducted into the Entourage Effect as an explanation for these phenomena in the context of cancer cell invasion before any conclusions can be drawn.

With the present significant focus on cannabis extracts and pure cannabinoids in the context of various pathologies, including malignancy, we may anticipate a better understanding of the complex pathways by which cannabinoids affect basic cell biology. This should prove extremely useful for the purpose of defining the situations where cannabinoids may be beneficial, where they may be contraindicated, and will also aid development of their potential as novel therapeutic agents.

In this review, the effects of cannabinoids on cancer cell migration, invasion, and metastatic spread have been discussed, with particular attention to the subtle concept of biased signaling. What becomes clear when reviewing the literature, is the variety of effects that cannabinoids can have on cancer cell invasion and metastasis. Various studies have seen contrasting increases and decreases in the metastatic potential of malignant cells when exposed to cannabinoid molecules, and biased signaling seems to account for a significant amount of this phenotypic diversity. Cannabinoid signaling in the context of cancer progression and development has been primarily investigated through three receptors, CB1, CB2, and GPR55, and each displays its own unique effects in various contexts. This is a rich and rewarding landscape for future investigation, through both discovery research and population studies reflecting on cannabis used.

## CONFLICT OF INTEREST

The authors declare no conflicts of interest.

## AUTHOR CONTRIBUTIONS

All authors conceived of, conducted the review of the literature, and wrote the manuscript. *Conceptualization; investigation; methodology; writing‐original draft; writing‐review & editing*, J.G.; *Conceptualization; investigation; methodology; writing‐review & editing*, J.B.

## ETHICS STATEMENT

Not applicable.

## Data Availability

Data sharing is not applicable to this article as no new data were created or analyzed in this study.
